# Manufacturing ultra-concentrated liquid feeds: Transitioning the aqueous solubility barrier of the feed amino acids cysteine and tyrosine

**DOI:** 10.1186/1753-6561-9-S9-P54

**Published:** 2015-12-14

**Authors:** Claudia Knack, Caroline Zessel, Christian Schultheiß, Doris Matheis, Christian Schild, Jörg von Hagen, Michael Rayner, Jochen Sieck

**Affiliations:** 1 Merck KGaA, Merck Millipore Division, Process Solutions, Pharm Chemicals Solutions R&D, Frankfurter Strasse 250, 64293 Darmstadt, Germany

## Background

Fed-batch cultivation is currently the most prominent process for the production of Biologics using mammalian cell lines such as CHO cells. Performance of fed-batch processes greatly depends on the feeding strategy. One important aspect of feeds is the nutrient concentration, which should be as high as possible. However, for some nutrients, there are solubility and/or stability limitations in aqueous solution. Thus, alternative systems for liquid feed manufacturing were considered. Deep Eutectic Solvents (DES) are salts in liquid state with a melting point under 100°C in the absence of water that form a eutectic system with a lower melting point than either of the individual components. The result is a highly concentrated, water free liquid system. A common component of DES is choline chloride (ChCl), a quaternary ammonium salt. For cell culture applications, ChCl is particularly well suited, since it is already widely used as cell culture medium component. We have experimentally manufactured deep eutectic solvents (DES) for application as cell culture feeds containing tyrosine and cysteine, which are challenging due to their weak solubility and/or stability in aqueous solution.

## Results

Various DES solutions were manufactured, which showed very different characteristics in terms of concentrations, pH, stability and viscosity. The precise mechanisms of DES formation and the underlying principles are poorly understood, making predictions of the DES properties very difficult. In general, the studied DES were highly viscous fluids. One of the manufactured Cys-ChCl DES was stable for 6 months. The following table (Table 1) shows the concentrations of cysteine and tyrosine in selected DES compared to a typical aqueous Cys/Tyr stock solution. The DES systems, including only crystal water, allows for dramatic increases in feed concentrations.

**Table 1:** Concentrations of pure cysteine and tyrosine in DES feeds compared to a typical Cys/Tyr feed.

Feed	Cysteine (% w/w)	Tyrosine (% w/w)
CHO Cys/Tyr stock solution	3.2	9.14
DES (ChCl + L-Cys HCl H_2_O, 1:1)	38.99	-
DES (ChCl + H_2_O + L-Tyr, 3:3:1)	-	26.21

**Figure 1:**
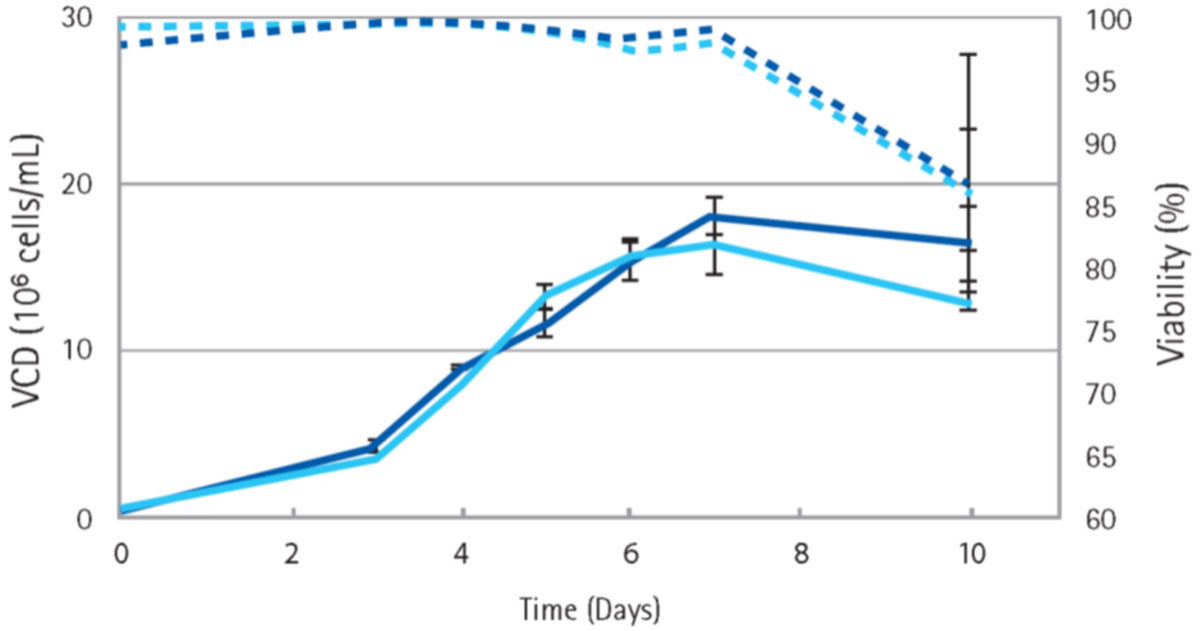
**Viable cell density and Viability during fed-batch cultivation of CHO cells using DES feeds compared to a typical aqueous Cys/Tyr feed solution**.

In a preliminary experiment for testing DES as cell culture feed, fed-batch cultivations were carried out comparing DES and a typical Cys/Tyr stock solution for Cys/Tyr addition. Figure 1 shows the results in terms of viable cell density (VCD) and viability. Viable Cell Density in DES fed cultures was slightly lower on day 10. Osmolality was slightly higher for the DES approach, as can be expected for addition of higher concentrated feeds (data not shown). Absolute mAb concentration was slightly lower due to lower VCD, but specific productivity was about similar (data not shown).

## Conclusions

We have successfully manufactured DES for use as cell culture feeds. L-cysteine hydrochloride monohydrate (L-Cys HCl H2O) and L-tyrosine hydrochloride (L-Tyr HCl) were used to form two DES with Choline Chloride. A range of temperatures and substance ratios to form a stable DES were tested.

Subsequently, the DES feeds were compared in a cell culture fed-batch process using a typical Cyr/Tyr CHO feed for comparison. Our preliminary results suggest that DES could be used as alternative cell culture feeds in the future. Possible advantages of such a strategy would be lower feed volumes required due to ultra-high feed concentration, no extra water (expect hydrate) diluting the content and thus less volume increase in bioreactors and improved bioreactor utilization.

However, there are many open points to study. It would be preferable to use one DES feed including both Cys/Tyr, but such a system showed to be far more complex to manufacture. Stability and bioavailability need to be studied in detail. Tests with additional cell lines, different feeding regimes etc. are ongoing. Even if these points can be clarified, it would be advisable to develop a completely new feeding strategy for DES to increase the performance with these feeds.

